# Prediction of Surgical Intervention in Acute Knee Trauma: A Focus on Threshold-Specific Performance and Clinical Decision Utility

**DOI:** 10.3390/diagnostics16111578

**Published:** 2026-05-22

**Authors:** Eun Byeol Choe, Joungeun Lee, Won-Kee Choi, Young Woo Seo, Sang Gyu Kwak

**Affiliations:** 1 Department of Emergency Medicine, Daegu Catholic University Hospital, Daegu 42472, Republic of Korea; ruaebd97@dcmc.co.kr (E.B.C.); zaiaz12815@dcmc.co.kr (J.L.); 2 Department of Orthopaedic Surgery, Daegu Catholic University School of Medicine, Daegu 42472, Republic of Korea; cwk10090@cu.ac.kr; 3 Department of Emergency Medicine, Daegu Catholic University School of Medicine, Daegu 42472, Republic of Korea; 4 Department of Medical Statistics, Daegu Catholic University School of Medicine, Duryugongwon-Ro 17-Gil 33, Nam-Gu, Daegu 42472, Republic of Korea

**Keywords:** acute knee trauma, decision curve analysis, machine learning, prediction model, risk stratification, surgical intervention

## Abstract

**Background**: Acute knee trauma is a common reason for emergency department visits, yet early identification of patients requiring surgical intervention remains challenging. Most existing prediction studies focus on discrimination metrics and provide limited guidance for clinical decision-making. **Methods**: We conducted a retrospective study of 905 patients presenting to the emergency department with acute knee trauma. Prediction models were developed using logistic regression, random forest, and extreme gradient boosting (XGBoost) based on routinely available clinical variables. Model performance was evaluated in terms of discrimination (AUROC, AUPRC), calibration, and clinical utility. Threshold-specific performance metrics and decision curve analysis were used to assess clinical applicability, and patients were stratified into risk groups based on predicted probabilities. **Results**: Among 905 patients, 163 (18.0%) underwent surgical intervention. Logistic regression and random forest demonstrated comparable performance (AUROC 0.748 and 0.744, respectively), whereas XGBoost showed lower discrimination (AUROC 0.632). Calibration was acceptable overall but less stable at higher predicted probabilities. Threshold-specific analysis demonstrated meaningful trade-offs between sensitivity and specificity across probability thresholds. Decision curve analysis showed that the model provided greater net benefit than default strategies within a threshold range of approximately 0.05–0.25. Risk stratification showed increasing surgical rates across risk groups, although the degree of separation was modest. **Conclusions**: Prediction models based on routinely available clinical variables can support early risk assessment in acute knee trauma. Their clinical usefulness depends on threshold-specific evaluation and decision-analytic approaches rather than overall performance metrics alone. These findings highlight the importance of interpreting prediction models within a clinical decision-making framework to facilitate real-world application.

## 1. Introduction

Acute knee trauma is a common reason for emergency department (ED) visits and a frequent musculoskeletal presentation in acute care practice. Early clinical decision-making in these patients is often challenging because injury mechanisms and severity are heterogeneous, whereas only a subset ultimately requires surgical intervention. This mismatch may contribute to inefficient resource utilization, unnecessary imaging, and delays in appropriate referral and management.

To improve diagnostic efficiency, clinical decision rules such as the Ottawa Knee Rule have been developed and extensively validated to guide the use of radiography in acute knee injuries [1,2]. These rules show excellent sensitivity for detecting fractures and have helped reduce unnecessary imaging. However, their primary role is diagnostic exclusion, and they do not address clinically meaningful downstream outcomes such as the need for surgical intervention. Accordingly, prediction models focused on the probability of surgical intervention may complement existing diagnostic decision tools by supporting subsequent management decisions, including specialist referral and advanced imaging. In routine practice, decisions regarding further evaluation, specialist referral, and possible surgical management therefore remain largely dependent on clinician judgment rather than objective early risk stratification.

Advanced imaging modalities, particularly magnetic resonance imaging (MRI), provide detailed structural assessment of soft tissue injuries, including anterior cruciate ligament and meniscal tears [3]. However, MRI is not routinely available in the ED setting, may delay decision-making, and does not directly provide a probabilistic estimate of surgical necessity. Because the need for surgical intervention is a clinically meaningful and actionable outcome that directly affects management planning and resource use, there is a clear unmet need for an early prediction tool that can identify patients at higher risk of requiring surgery using information available at initial presentation.

Recent studies and systematic reviews have shown increasing interest in prediction models and machine learning approaches in orthopedic surgery and trauma [4,5,6,7,8,9]. Although these models have demonstrated promising discriminatory performance, their methodological quality and clinical applicability remain limited [4,7]. In particular, many prior studies have emphasized discrimination metrics such as the area under the receiver operating characteristic curve (AUROC), whereas calibration and clinical usefulness have received less attention despite their importance for real-world implementation [10,11]. Reporting quality and risk of bias also remain important concerns, as highlighted by frameworks such as TRIPOD and PROBAST [12,13]. Moreover, while machine learning methods may capture complex relationships among predictors, their clinical usefulness and interpretability require careful evaluation [14,15]. Beyond orthopedic applications, machine learning and explainable artificial intelligence approaches have also been increasingly applied across diverse healthcare domains, including chronic disease prediction and medical imaging analysis. Recent studies have demonstrated the potential utility of explainable machine learning models for early detection of type 2 diabetes [16]. These developments highlight the expanding role of artificial intelligence in supporting clinical decision-making across multiple medical fields.

Beyond predictive accuracy, effective clinical decision-making requires explicit consideration of risk thresholds, as described in the threshold model of decision-making [17]. Clinicians must determine whether the estimated probability of an outcome is high enough to justify further investigation or intervention. However, most existing prediction studies do not provide threshold-specific performance measures or clinically actionable decision thresholds, limiting their practical use. Decision-analytic approaches such as decision curve analysis can address this gap by quantifying net benefit across a range of threshold probabilities and linking model performance to clinical consequences [18]. In acute knee trauma, different thresholds may correspond to distinct strategies, such as safely ruling out the need for surgery at lower thresholds or prioritizing early referral and advanced imaging at higher thresholds. Accordingly, evaluating model performance across clinically relevant thresholds is essential for translating prediction models into practical decision support tools.

The primary aim of this study was to develop and evaluate prediction models for identifying patients with acute knee trauma who are likely to require surgical intervention using routinely available clinical variables obtained at ED presentation. We compared conventional logistic regression with machine learning algorithms and performed a comprehensive assessment of model performance, including discrimination, calibration, and clinical utility. In addition, we explicitly evaluated threshold-specific performance and decision-analytic utility to identify clinically actionable decision thresholds and translate these into interpretable risk stratification for real-world use. By focusing on threshold-based clinical applicability rather than predictive accuracy alone, this study seeks to bridge the gap between statistical prediction and actionable decision support in acute knee trauma care. Unlike many previous prediction studies that primarily emphasized overall predictive performance or postoperative outcomes, the present study specifically focused on clinically interpretable threshold-based evaluation and decision-analytic utility for predicting subsequent surgical intervention in acute knee trauma using routinely available emergency department variables to facilitate practical clinical decision-making. Rather than replacing existing clinical assessment pathways or established diagnostic decision rules, the proposed model is intended to function as a complementary decision-support tool that may assist secondary risk stratification following the initial evaluation of patients with acute knee trauma.

## 2. Materials and Methods

### 2.1. Study Design and Setting

This retrospective observational study was conducted using data from patients who presented with acute knee trauma to the emergency department (ED) of a tertiary care hospital. The aim of this study was to develop and evaluate prediction models for identifying patients who are likely to require surgical intervention at the time of initial presentation. The study was approved by the Institutional Review Board of Daegu Catholic University Medical Center (IRB No. DCUMC-2026-04-014; date of approval: 8 April 2026), and the requirement for informed consent was waived due to the retrospective nature of the study. Clinical variables were extracted from electronic medical records and included routinely available information obtained during the initial emergency department evaluation.

### 2.2. Study Population

**Figure 1:**
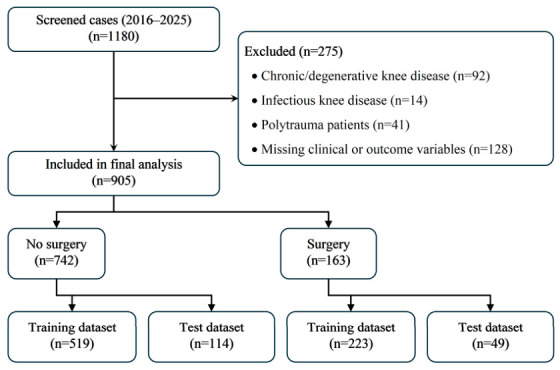
Study flow diagram of patient selection and dataset partitioning. Patients presenting to the emergency department with acute knee trauma were screened according to predefined inclusion and exclusion criteria. The final study population was categorized by surgical intervention and split into training and test datasets for model development and validation.

Patients were eligible for inclusion if they presented to the emergency department (ED) with acute knee trauma and underwent standard clinical evaluation, including history taking, physical examination, and radiographic assessment. Patients were excluded if they had chronic or degenerative knee disease, infectious knee conditions, polytrauma, or missing clinical or outcome variables, as detailed in Figure 1. A complete-case analysis was performed, and patients with incomplete predictor or outcome data were excluded prior to model development. Because the proportion of missing data was relatively small, no additional imputation procedures were applied. For patients with multiple ED visits during the study period, only the first visit was included in the analysis.

### 2.3. Outcome Definition

The primary outcome was surgical intervention related to the index knee injury within 90 days after the ED visit. Surgical intervention was defined as any operative procedure performed for the management of the injury after the ED visit. This outcome was selected as a clinically meaningful and actionable endpoint that directly influences patient management, including decisions regarding referral, advanced imaging, and resource utilization.

### 2.4. Predictor Variables

Predictor variables were selected based on clinical relevance and availability at the time of emergency department (ED) presentation. No univariable screening or stepwise variable selection procedures were performed. The study aimed to evaluate the predictive performance of routinely available clinical variables within a clinically interpretable framework. These included demographic characteristics (age, sex, and body mass index), injury-related factors (mechanism of injury), and clinical findings such as pain score measured using a numeric rating scale, presence of knee swelling, and limitation of range of motion. Triage severity was assessed using the Korean Triage and Acuity Scale (KTAS). Radiographic findings were defined based on plain radiography and included fractures, dislocations, and other clinically relevant acute traumatic abnormalities, as documented in formal radiology reports. Detailed definitions are provided in the Supplementary Materials.

### 2.5. Model Development

Prediction models were developed using logistic regression, random forest, and extreme gradient boosting (XGBoost). The dataset was randomly divided into training and test sets in a 7:3 ratio using stratified sampling according to the outcome. Logistic regression was included as a reference model because of its interpretability and widespread use in clinical research, whereas machine learning models were applied to explore potential advantages in capturing complex relationships among predictors. XGBoost was selected because it is a well-established algorithm for structured tabular clinical data and has shown robust performance in previous clinical prediction studies. Deep learning approaches were not applied because the present study used a relatively moderate-sized dataset composed of routinely collected structured clinical variables rather than high-dimensional data modalities such as medical imaging, and this data structure was unlikely to support stable performance advantages over conventional machine learning approaches. A total of 163 surgical events were available, and the final models included 9 predictor variables, resulting in an approximate event-to-variable ratio (EPV) of 18.1. This EPV was considered acceptable for prediction model development and was expected to reduce the risk of model overfitting. Hyperparameters for the machine learning models were optimized using 5-fold cross-validation within the training dataset (details are provided in the Supplementary Materials), and internal validation metrics were obtained from the same cross-validation procedure. The independent test dataset was not used during model development or hyperparameter optimization and was reserved exclusively for final model evaluation to minimize the risk of information leakage and over-optimization. As an additional sensitivity analysis, temporal validation was performed by training the final logistic regression model using cases from 2016–2022 and evaluating performance in a temporally independent cohort from 2023–2025.

### 2.6. Model Performance Evaluation

Model performance was assessed using multiple complementary metrics. Discrimination was evaluated using the area under the receiver operating characteristic curve (AUROC) and the area under the precision–recall curve (AUPRC). Calibration was assessed using the Brier score and calibration plots comparing predicted and observed probabilities. These metrics were selected to provide a comprehensive evaluation of model performance: AUROC reflects overall discriminative ability, AUPRC is particularly informative in imbalanced datasets, and calibration assesses the accuracy of predicted probabilities, consistent with established recommendations for statistical analysis and reporting of prediction models [12,18,19,20]. Given the moderate class imbalance (18.0% surgical intervention rate), no additional resampling or class-weighting techniques were applied. Instead, model performance was evaluated using metrics appropriate for imbalanced data, including the area under the precision–recall curve (AUPRC).

### 2.7. Threshold-Specific Performance Analysis

To enhance clinical applicability, model performance was further evaluated across a range of predefined probability thresholds. For each threshold, sensitivity, specificity, positive predictive value, and negative predictive value were calculated. These analyses were conducted to identify clinically relevant thresholds that could support decision-making, such as safely ruling out the need for surgical intervention at low-risk thresholds or prioritizing further evaluation and referral at higher-risk thresholds. The selected thresholds were intended to represent clinically meaningful ranges for exploratory decision-making rather than fixed intervention thresholds. Lower thresholds were considered suitable for rule-out strategies aimed at minimizing missed surgical cases, whereas higher thresholds may support prioritization of specialist referral or advanced imaging. In addition, patients were categorized into low (<0.10), intermediate (0.10–0.29), and high-risk (≥0.30) groups based on predicted probabilities derived from the final model.

### 2.8. Decision Curve Analysis

Decision curve analysis was performed for the final prediction model to evaluate its clinical utility by quantifying net benefit across a range of threshold probabilities. This approach allows assessment of whether the use of the model provides greater clinical benefit compared with default strategies, such as treating all patients or none, thereby linking model performance to clinical decision-making.

### 2.9. Model Interpretability

SHapley Additive exPlanations (SHAP) were used to quantify the contribution of each predictor to model predictions. SHAP values were used to identify important variables and to characterize the magnitude and direction of their influence on the predicted probability of surgical intervention. This approach enhances model interpretability and transparency by providing insight into how individual predictors contribute to model outputs.

### 2.10. Statistical Analysis

Continuous variables are presented as mean ± standard deviation and were compared using Student’s *t*-test. Categorical variables are presented as counts (percentages) and were compared using the chi-square test or Fisher’s exact test, as appropriate. Standardized mean differences (SMDs) were calculated to assess the magnitude of differences between groups, with an absolute SMD > 0.1 indicating meaningful imbalance. All tests were two-sided, and a *p*-value < 0.05 was considered statistically significant. All analyses were performed using Python (version 3.12). The analysis code is publicly available at: https://github.com/sanggyu3939/knee-surgery-prediction (accessed on 20 April 2026).

## 3. Results

### 3.1. Patient Characteristics

A total of 905 patients with acute knee trauma were included in the analysis, of whom 163 (18.0%) underwent surgical intervention. Baseline characteristics of the study population are presented in Table 1. Patients who underwent surgery were older than those who did not (52.87 ± 20.00 vs. 47.99 ± 19.02 years, *p* = 0.005) and had higher pain scores (5.87 ± 1.51 vs. 5.28 ± 1.50, *p* < 0.001). Knee swelling and limitation of range of motion were more frequent in the surgery group, with large standardized mean differences (SMD = 0.703 and 0.575, respectively). Injury mechanism differed between groups (*p* < 0.001), with traffic-related injuries more common among patients who underwent surgery (21.5% vs. 6.2%, SMD = 0.454). Abnormal findings on plain radiography were also more frequent in the surgery group (29.4% vs. 15.2%, SMD = 0.346). Several variables showed meaningful between-group differences (SMD > 0.1).

### 3.2. Model Performance

**Figure 2:**
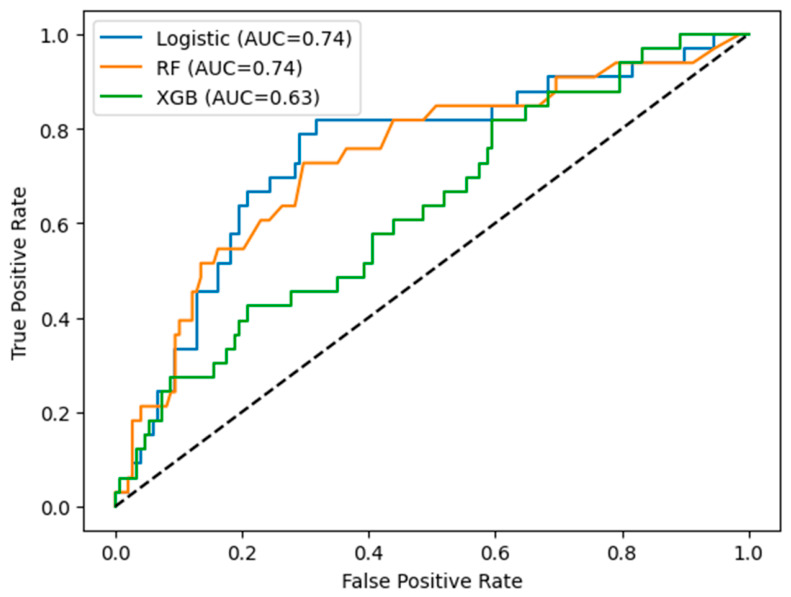
Receiver operating characteristic (ROC) curves of prediction models. The ROC curves compare the discriminative performance of logistic regression, random forest, and XGBoost models in the independent test set. The dashed diagonal line represents the performance of a non-informative classifier. Logistic regression and random forest demonstrated similar performance, whereas XGBoost showed inferior discrimination.

**Figure 3:**
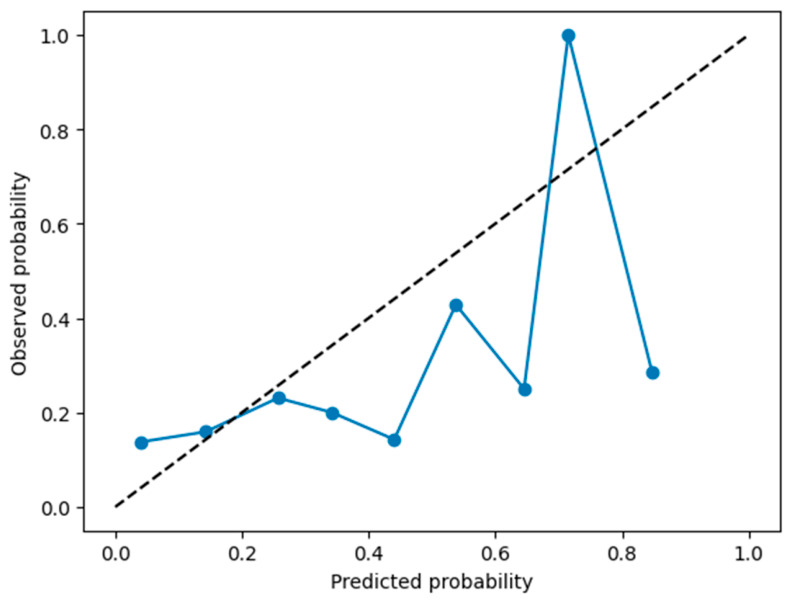
Calibration plot of the final prediction model. The plot compares predicted probabilities with observed outcomes across risk deciles in the independent test set. The dashed diagonal line represents perfect calibration. While the model shows reasonable agreement in lower probability ranges, some deviation is observed in higher probability ranges, indicating imperfect calibration at extreme predictions.

To provide a comprehensive assessment of predictive performance and clinical applicability, discrimination, calibration, threshold-specific performance, and decision-analytic utility were evaluated using complementary metrics. The performance of the prediction models is summarized in Table 2, and the receiver operating characteristic (ROC) curves are shown in Figure 2. Logistic regression and random forest demonstrated similar discrimination, with AUROC values of 0.748 and 0.744, respectively. XGBoost showed lower discrimination (AUROC 0.632). A similar pattern was observed for AUPRC, with comparable performance between logistic regression and random forest and lower values for XGBoost. Calibration performance, assessed using the Brier score, was comparable between logistic regression and random forest, whereas XGBoost showed poorer calibration. Calibration plots (Figure 3) demonstrated reasonable agreement between predicted and observed probabilities for logistic regression and random forest, with some deviation at higher predicted probabilities. Overall, logistic regression and random forest showed similar performance across multiple evaluation metrics, whereas XGBoost performed less favorably. For the final logistic regression model, calibration assessment showed a calibration intercept of −0.309 (95% CI, −0.989 to 0.395) and a calibration slope of 0.864 (95% CI, 0.457 to 1.418), suggesting acceptable overall calibration without strong evidence of systematic overestimation or severe overfitting. Additional temporal validation was performed using earlier cases for model development and a temporally independent cohort for validation. Temporal validation demonstrated preserved discrimination performance, with an AUROC of 0.698 (95% CI, 0.615–0.782), an AUPRC of 0.408 (95% CI, 0.292–0.555), and a Brier score of 0.136 (95% CI, 0.108–0.166). Calibration assessment showed a calibration intercept of −0.456 and a calibration slope of 0.606, suggesting some reduction in calibration stability over time. Final model selection was based on an integrated assessment intended to balance discrimination performance, calibration characteristics, threshold-specific clinical utility, and interpretability rather than relying solely on discrimination metrics such as AUROC values. Based on these considerations, logistic regression was selected as the final prediction model for threshold-specific evaluation, decision curve analysis, and risk stratification.

### 3.3. Threshold-Specific Performance

Model performance across different probability thresholds is summarized in Table 3. At a threshold of 0.10, the model showed high sensitivity (0.606) and negative predictive value (0.869). As the threshold increased, sensitivity decreased and specificity increased. At a threshold of 0.20, sensitivity and specificity were 0.455 and 0.736, respectively. At higher thresholds (≥0.30), specificity increased further. These results illustrate the trade-offs in model performance across different thresholds.

### 3.4. Decision Curve Analysis

**Figure 4:**
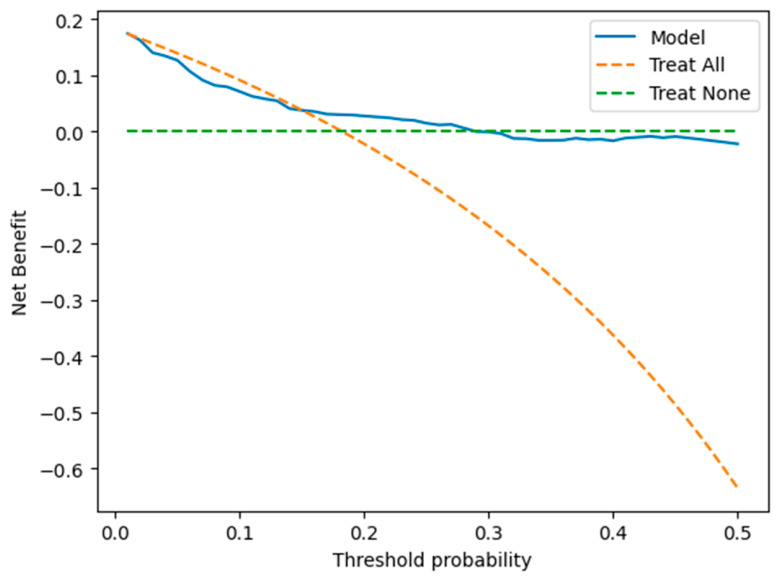
Decision curve analysis of the final prediction model. The net benefit of the prediction model is compared with the treat-all and treat-none strategies across a range of threshold probabilities. The prediction model demonstrates a higher net benefit than both strategies within a clinically relevant range of threshold probabilities, indicating potential clinical utility.

Decision curve analysis (Figure 4) showed that the prediction model provided greater net benefit than the default strategies (treat-all and treat-none) across a range of threshold probabilities. The model demonstrated the highest net benefit within the threshold range of approximately 0.05 to 0.25. Within this range, the net benefit of the model exceeded that of the default strategies.

### 3.5. Risk Stratification

Patients were stratified into risk groups based on predicted probabilities (Table 4). The observed surgical rates increased across risk groups, from 13.8% in the low-risk group to 29.7% in the high-risk group. These findings suggest that the model may help distinguish between relatively different levels of risk, although the degree of separation was modest.

### 3.6. Model Interpretability

Feature importance analysis using SHAP values (Supplementary Figure S3) identified several clinically important predictors of surgical intervention, including age, pain score, knee swelling, limitation of range of motion, and radiographic abnormalities. Higher pain scores, the presence of knee swelling or range of motion limitation, and abnormal radiographic findings were generally associated with increased predicted probabilities of surgical intervention. These findings were clinically consistent with factors reflecting greater injury severity and functional impairment. Additional performance evaluations, including precision–recall curves and threshold-dependent performance trends, are shown in Supplementary Figures S1 and S2.

## 4. Discussion

In this study, we developed and evaluated prediction models for surgical intervention in patients presenting to the emergency department with acute knee trauma using routinely available clinical variables. Several important findings emerged from this study. First, logistic regression and random forest demonstrated comparable discriminative performance, whereas XGBoost showed lower discrimination and less favorable calibration characteristics. Second, calibration assessment suggested generally acceptable agreement between predicted and observed probabilities, although calibration instability was observed at higher predicted probabilities. Third, threshold-specific evaluation and decision curve analysis demonstrated that the clinical usefulness of prediction models depends not only on overall discrimination performance but also on threshold-dependent trade-offs and clinical decision consequences. Finally, the proposed model was designed as a complementary risk stratification tool intended to support, rather than replace, existing clinical decision-making processes following initial evaluation in acute knee trauma.

These findings suggest that more complex machine learning models did not demonstrate superior performance in this study when using routinely collected clinical variables, consistent with previous observations in clinical prediction research [13]. Previous orthopedic and trauma prediction studies have reported variable discrimination performance depending on the target population, predictor composition, and clinical outcome definitions [7,8,9,10,11]. Direct comparison across studies remains challenging because some models were developed for postoperative outcomes, imaging findings, or revision risk rather than prediction of subsequent surgical intervention after acute trauma presentation. This may be explained by the relatively limited number of predictors and the structured nature of the data, where complex nonlinear modeling may not provide additional benefit over simpler models.

A key contribution of this study is the explicit evaluation of model performance across a range of probability thresholds, rather than relying solely on global performance metrics. While most previous studies have focused primarily on overall discrimination metrics such as AUROC, these measures do not directly inform clinical decision-making [10]. In contrast, calibration assessment, threshold-specific performance reporting, and decision-analytic approaches such as decision curve analysis remain comparatively under-reported despite their importance for real-world clinical implementation. Our results show that model performance varies meaningfully across thresholds, highlighting the importance of considering threshold-dependent trade-offs between sensitivity and specificity. This approach is aligned with the threshold-based decision-making framework described by Pauker and Kassirer [17].

Decision curve analysis further demonstrated that the prediction model provided greater net benefit than default strategies across a range of threshold probabilities. Decision curve analysis incorporates the relative consequences of false-positive and false-negative decisions and has been recommended as a method to evaluate the clinical usefulness of prediction models [18]. In our study, the model showed the highest net benefit within a threshold range of approximately 0.05 to 0.25. Rather than proposing a single optimal threshold, these findings illustrate how different thresholds may be applied depending on the clinical context. The choice of threshold in clinical practice may depend on the relative consequences of false-positive and false-negative decisions, available healthcare resources, and clinician preference. Therefore, the thresholds evaluated in this study should be interpreted as clinically exploratory rather than universally applicable cutoff values. In practice, low predicted probabilities may support less intensive immediate evaluation, whereas higher probabilities may prompt early referral or consideration of advanced imaging. From a practical perspective, the proposed model may serve as a secondary risk stratification tool following initial emergency department assessment, potentially assisting clinicians in identifying patients who may benefit from closer orthopedic evaluation, early specialist referral, or consideration of advanced imaging. Importantly, the proposed model is not intended to replace existing clinical decision tools such as the Ottawa Knee Rule. Rather, it may serve a complementary role by providing probabilistic risk assessment for subsequent surgical intervention after the initial diagnostic evaluation has been completed. Direct comparison with existing clinical decision rules is challenging because most available tools were developed for fracture exclusion or imaging guidance rather than prediction of subsequent surgical intervention. In addition, previously published prediction studies for surgical intervention in knee disorders have used different patient populations, predictor variables, and outcome definitions, limiting direct application and like-for-like comparison within the present cohort. Future studies using standardized datasets and harmonized outcome definitions may enable more robust head-to-head benchmarking against existing approaches. Nevertheless, the discrimination performance observed in the present study was comparable to that reported in previous orthopedic prediction studies using structured clinical variables. From a practical perspective, the observed net benefit suggests that the model may help reduce unnecessary advanced evaluation or specialist referral in selected low-risk patients while maintaining identification of patients who may ultimately require surgical intervention. For example, at a threshold probability of 0.20, the logistic regression model was associated with an estimated reduction of approximately 39 unnecessary referrals or advanced evaluations per 100 patients compared with a treat-all strategy. However, the precise clinical impact of this strategy should be further evaluated in prospective implementation studies.

Risk stratification based on predicted probabilities showed increasing surgical rates across risk groups, suggesting that the model was able to stratify patients according to their relative likelihood of undergoing surgical intervention within 90 days after emergency department presentation, although the degree of separation was modest. The proposed risk groups were intended to support relative risk assessment and early clinical triage rather than to define definitive treatment categories. In acute care settings, even moderate differences in predicted risk may assist decisions regarding further evaluation, specialist referral, or advanced imaging when only limited information is available at initial presentation [5,21].

Interpretability analysis using SHAP values identified clinically relevant predictors, including age, pain score, knee swelling, range of motion limitation, and radiographic abnormalities. The identified predictors were also clinically plausible and reflected factors commonly considered during early assessment of acute knee trauma in routine emergency care. The use of explainable machine learning techniques has been increasingly emphasized to improve transparency and trust in clinical prediction models [1,4].

This study has several strengths. First, we evaluated both traditional statistical and machine learning models using the same dataset and predictor variables, enabling a fair comparison. Second, multiple aspects of model performance were assessed, including discrimination, calibration, and decision curve analysis, consistent with recommended frameworks for prediction model evaluation [10,13]. Third, we emphasized threshold-specific evaluation, which directly connects model outputs to clinical decision-making and addresses a key limitation of prior prediction studies. This integrated evaluation framework may provide more clinically meaningful interpretation of prediction model performance compared with reliance on discrimination metrics alone.

However, several limitations should be considered. First, this was a retrospective study conducted at a single center, which may limit generalizability, and selection bias may exist as the study population consisted of patients presenting to a single emergency department. Second, although routinely available clinical variables were used to enhance applicability, unmeasured confounding cannot be excluded. Although the event-to-variable ratio was considered acceptable in the present study, larger datasets may further improve the robustness and stability of machine learning models, particularly for more complex algorithms. Third, calibration was less stable at higher predicted probabilities, suggesting caution when interpreting extreme risk estimates. This instability may reflect the relatively limited number of high-risk cases and possible overestimation of risk at the upper end of the probability spectrum. Clinically, high predicted probabilities should therefore be interpreted as supportive risk indicators rather than definitive indications for surgical intervention. Accordingly, high predicted probabilities may be most appropriately used to support closer clinical assessment, consideration of advanced imaging, or early specialist referral rather than as standalone criteria for operative decision-making. Future studies using larger and externally validated datasets may allow recalibration and further optimization of model calibration. In the meantime, the use of multiple complementary evaluation methods, including threshold-specific performance analysis and decision curve analysis, may help improve the robustness and clinical interpretability of prediction model assessment beyond discrimination metrics alone. Fourth, although additional temporal validation demonstrated moderate preservation of model performance in a temporally independent cohort, geographic external validation was not performed. Therefore, future studies using multicenter and geographically independent datasets are required to further evaluate the robustness, transportability, and generalizability of the model across different clinical settings, as recommended in current reporting guidelines [13]. Temporal validation may be particularly important because clinical management strategies, referral patterns, and surgical decision-making processes may evolve over time, potentially influencing model calibration and predictive performance. Temporal validation demonstrated moderate preservation of discrimination performance in a temporally independent cohort, although some reduction in calibration stability was observed. These findings suggest that periodic recalibration and external validation may be important before implementation across evolving clinical environments and healthcare systems. These factors may contribute to inter-institutional variability and could limit direct application of the model across healthcare systems with different clinical workflows and resource availability. Future multicenter and externally validated studies are needed to further assess model robustness, transportability, and real-world clinical applicability. Finally, although threshold-based evaluation and decision curve analysis provide insight into potential clinical utility, the actual impact of implementing this model in clinical practice remains to be determined. In addition, the outcome of surgical intervention within 90 days may partially reflect clinician judgment, patient preference, and healthcare accessibility rather than injury severity alone. Therefore, some heterogeneity in the outcome definition may have been unavoidable and could influence model transportability across different clinical settings. In addition, the selection of appropriate probability thresholds in real-world settings may require further clinical validation and consensus. In addition, inappropriate reliance on model predictions without sufficient clinical assessment may introduce safety concerns. For example, incorrect interpretation of low predicted risk could potentially delay referral or further evaluation in patients who may ultimately require surgical intervention. Therefore, the proposed model should be used as a supportive decision aid rather than a replacement for clinical judgment. Overall, our findings emphasize the importance of interpreting prediction models within a clinical decision-making framework rather than relying solely on traditional performance metrics.

## 5. Conclusions

In this study, we developed and evaluated prediction models for surgical intervention in patients with acute knee trauma using routinely available clinical variables obtained at emergency department presentation. Logistic regression and random forest demonstrated comparable performance, whereas XGBoost showed less favorable discrimination and calibration. The clinical usefulness of the models depended on threshold-specific evaluation and decision-analytic assessment rather than overall performance metrics alone. Although internal validation demonstrated potential clinical utility across a range of threshold probabilities, external validation was not performed. Future multicenter and externally validated studies are needed to further improve assessment of model robustness, transportability, and real-world clinical applicability. The proposed model may function as a complementary risk stratification tool following initial clinical assessment rather than as a standalone replacement for existing decision-making processes.

## Figures and Tables

**Table 1:** Baseline characteristics of the study population according to surgical intervention.

Variable	Total (n = 905)	Non-Surgery (n = 742)	Surgery (n = 163)	*p*-Value	SMD
Age, years	48.87 ± 19.28	47.99 ± 19.02	52.87 ± 20.00	0.005	0.250
Sex				0.093	
Male	552 (61.0)	443 (59.7)	109 (66.9)		0.149
Female	353 (39.0)	299 (40.3)	54 (33.1)		0.149
BMI, kg/m^2^	25.28 ± 3.86	25.38 ± 3.79	24.83 ± 4.14	0.119	0.139
Injury mechanism				<0.001	
Fall	307 (33.9)	266 (35.8)	41 (25.2)		0.234
Traffic accident	81 (9.0)	46 (6.2)	35 (21.5)		0.454
Twisting injury	169 (18.7)	152 (20.5)	17 (10.4)		0.281
Sports injury	274 (30.3)	216 (29.1)	58 (35.6)		0.139
Direct blow	74 (8.2)	62 (8.4)	12 (7.4)		0.037
Pain score (NRS)	5.38 ± 1.52	5.28 ± 1.50	5.87 ± 1.51	<0.001	0.390
Knee swelling				<0.001	
No	546 (60.3)	492 (66.3)	54 (33.1)		0.703
Yes	359 (39.7)	250 (33.7)	109 (66.9)		0.703
ROM limitation				<0.001	
No	576 (63.6)	509 (68.6)	67 (41.1)		0.575
Yes	329 (36.4)	233 (31.4)	96 (58.9)		0.575
KTAS level				0.101	
Level 2	28 (3.1)	20 (2.7)	8 (4.9)		0.116
Level 3	209 (23.1)	163 (22.0)	46 (28.2)		0.145
Level 4	468 (51.7)	395 (53.2)	73 (44.8)		0.170
Level 5	200 (22.1)	164 (22.1)	36 (22.1)		0.000
X-ray abnormality				<0.001	
No	744 (82.2)	629 (84.8)	115 (70.6)		0.346
Yes	161 (17.8)	113 (15.2)	48 (29.4)		0.346

Values are presented as mean ± standard deviation for continuous variables and number (percentage) for categorical variables. All *p*-values were calculated using Student’s *t*-test for continuous variables and the chi-square test for categorical variables, as appropriate. Standardized mean differences (SMDs) were calculated to assess the magnitude of differences between groups. BMI, body mass index; NRS, numeric rating scale; ROM, range of motion; KTAS, Korean Triage and Acuity Scale.

**Table 2:** Performance of prediction models using cross-validation and independent test set.

Model	Logistic Regression	Random Forest	XGBoost
Cross-validation			
AUROC	0.769 ± 0.050	0.739 ± 0.065	0.735 ± 0.049
AUPRC	0.462 ± 0.051	0.494 ± 0.061	0.397 ± 0.051
Brier score	0.122 ± 0.006	0.125 ± 0.011	0.136 ± 0.010
Test set			
AUROC	0.748 (0.642–0.844)	0.744 (0.635–0.843)	0.632 (0.522–0.730)
AUPRC	0.404 (0.272–0.565)	0.423 (0.286–0.609)	0.273 (0.179–0.427)
Brier score	0.133 (0.103–0.164)	0.131 (0.100–0.164)	0.162 (0.122–0.204)

Model performance in cross-validation is presented as mean ± standard deviation across 5 folds. Test set performance was evaluated on an independent hold-out dataset. Discrimination was assessed using AUROC and AUPRC, and calibration was evaluated using the Brier score. Lower Brier scores indicate better model calibration. For test set performance, 95% confidence intervals were estimated using bootstrap resampling. AUROC, area under the receiver operating characteristic curve; AUPRC, area under the precision–recall curve.

**Table 3:** Diagnostic performance of the final model at selected probability thresholds.

Threshold	Sensitivity	Specificity	PPV	NPV
0.1	0.606	0.581	0.244	0.869
0.15	0.515	0.669	0.258	0.861
0.2	0.455	0.736	0.278	0.858
0.25	0.303	0.797	0.25	0.837
0.3	0.303	0.845	0.303	0.845
0.4	0.152	0.905	0.263	0.827
0.5	0.091	0.939	0.25	0.822

Diagnostic performance was evaluated across selected probability thresholds using the final prediction model. Sensitivity, specificity, positive predictive value (PPV), and negative predictive value (NPV) were calculated based on the independent test set. Thresholds were selected to represent clinically relevant ranges for rule-out and rule-in decision-making.

**Table 4:** Risk stratification based on predicted probability of surgical intervention.

Risk Group	N	Observed Surgery Rate (%)	Median Predicted Probability
Low	87	13.8	0.043
Intermediate	57	17.5	0.154
High	37	29.7	0.515

Patients were stratified into three risk groups based on predicted probabilities from the final model. The low-risk group was defined as predicted probability <0.10, intermediate-risk as 0.10–0.29, and high-risk as ≥0.30. Observed surgery rates were calculated within each group using the independent test set.

## Data Availability

The data presented in this study are available upon request from the corresponding author under ethical and legal restrictions related to patient confidentiality.

## Supplementary Materials

The following supporting information can be downloaded at: https://www.mdpi.com/article/10.3390/diagnostics16111578/s1, Figure S1: Precision–recall curves of prediction models; Figure S2: Threshold-dependent performance trends of the final model; Figure S3: SHAP summary plot for model interpretability; Supplementary Methods: Hyperparameter optimization and model development procedures.

## Abbreviations

The following abbreviations are used in this manuscript:

AUPRC	Area under the precision–recall curve
AUROC	Area under the receiver operating characteristic curve
BMI	Body mass index
CI	Confidence interval
DCA	Decision curve analysis
ED	Emergency department
KTAS	Korean Triage and Acuity Scale
ML	Machine learning
MRI	Magnetic resonance imaging
NRS	Numeric rating scale
NPV	Negative predictive value
PPV	Positive predictive value
ROC	Receiver operating characteristic
SHAP	SHapley Additive exPlanations
SMD	Standardized mean difference
XGBoost	Extreme gradient boosting
TRIPOD	Transparent Reporting of a multivariable prediction model for Individual Prognosis or Diagnosis
PROBAST	Prediction model Risk Of Bias ASsessment Tool
