# Supplementary material for: Prediction of Surgical Intervention in Acute Knee Trauma: A Focus on Threshold-Specific Performance and Clinical Decision Utility
Source: Diagnostics (Basel). 2026 May 22;16(11):1578. doi: 10.3390/diagnostics16111578 (PMC13257426; doi:10.3390/diagnostics16111578)
Supplement: Supplementary file 1 [file diagnostics-16-01578-s001.zip › supplementary materials.pdf]

Supplementary Materials

Supplementary Methods

Hyperparameter tuning

Hyperparameters for the machine learning models were optimized using cross-validation within the training dataset. For the random forest model, key hyperparameters included the number of trees (n_estimators), maximum tree depth (max_depth), and minimum number of samples required to split a node (min_samples_split). For the XGBoost model, hyperparameters included the number of boosting rounds (n_estimators), learning rate (eta), maximum tree depth (max_depth), subsampling ratio (subsample), and column sampling ratio (colsample_bytree). A grid search strategy was applied to identify optimal hyperparameter combinations. Model performance was evaluated using 5-fold cross-validation within the training dataset, and the set of hyperparameters yielding the best average performance was selected for the final model. All hyperparameter tuning procedures were conducted using Python libraries such as scikit-learn and XGBoost. A fixed random seed was used to ensure reproducibility of the model development process.

Internal validation

Internal validation was performed using 5-fold cross-validation within the training dataset. The dataset was randomly partitioned into five subsets, and in each iteration, four subsets were used for training and one for validation. This process was repeated five times so that each subset served as a validation set once. The average performance across folds was used to assess model stability and reduce the risk of overfitting. Final model performance was subsequently evaluated on an independent test dataset that was not used during model training or hyperparameter tuning.

Definition of radiographic abnormalities

Radiographic abnormalities were defined based on formal radiology reports interpreted by board-certified radiologists at the time of ED presentation. Abnormal findings included fractures, dislocations, and other clinically relevant acute traumatic structural abnormalities. Findings clearly unrelated to the acute traumatic event (e.g., chronic degenerative changes) were not considered abnormal.

Supplementary figure legends

Supplementary Figure S1. Precision–recall curves comparing the performance of logistic regression, random forest, and XGBoost models in the independent test set.

Supplementary Figure S2. Threshold-dependent performance curves of the final prediction model. Sensitivity, specificity, positive predictive value (PPV), and negative predictive value (NPV) are plotted across a range of probability thresholds. As the threshold increases, sensitivity decreases while specificity increases, demonstrating a trade-off between rule-out and rule-in strategies. The transition zone between thresholds of approximately 0.20 and 0.30 reflects a transition from sensitivity-dominant to specificity-dominant classification.

Supplementary Figure S3. SHAP summary plot of the final prediction model (top 10 features). The plot shows the top 10 most important features ranked by mean absolute SHAP values. Each point represents an individual patient, with the x-axis indicating the SHAP value (impact on model output). Red indicates higher feature values and blue indicates lower feature values, illustrating how each feature contributes to the predicted probability of surgical intervention.
