# Supplementary figures and images for: Prediction of Surgical Intervention in Acute Knee Trauma: A Focus on Threshold-Specific Performance and Clinical Decision Utility
Source: Diagnostics (Basel). 2026 May 22;16(11):1578. doi: 10.3390/diagnostics16111578 (PMC13257426; doi:10.3390/diagnostics16111578)

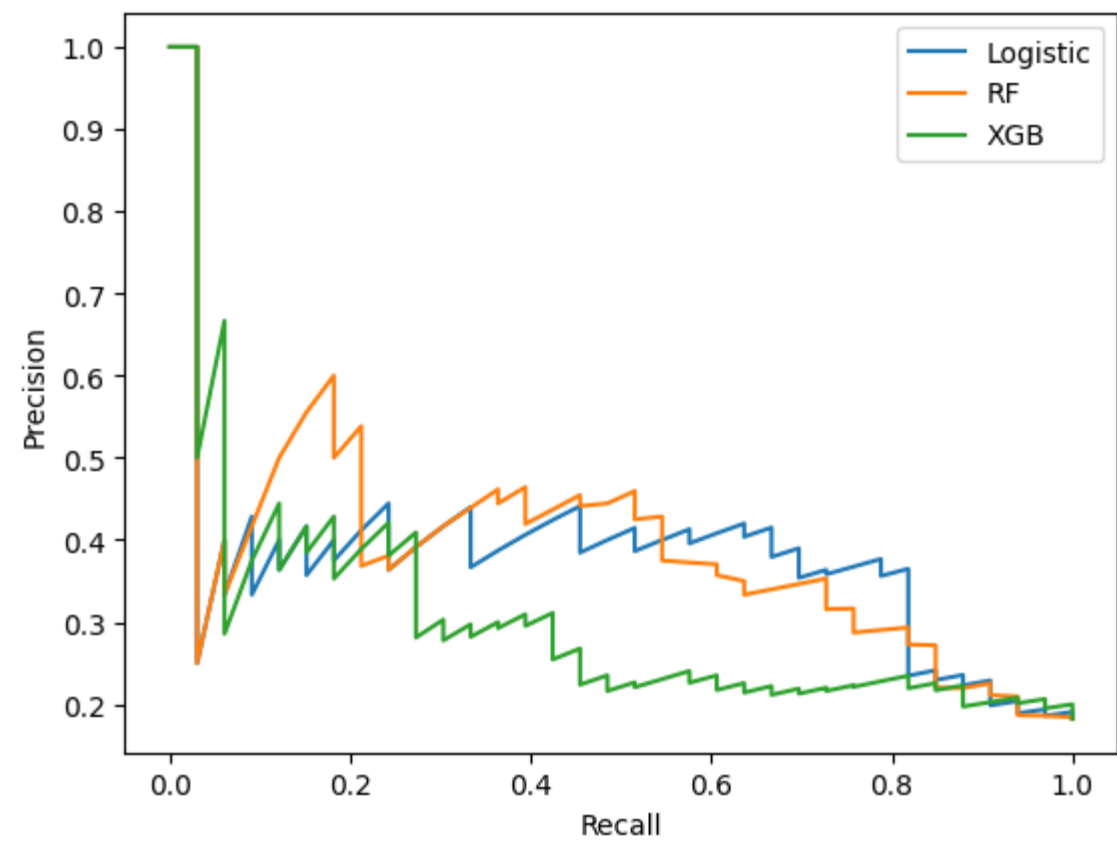

Supplement: Supplementary file 1 [file diagnostics-16-01578-s001.zip › Suuplementary Figure S1.pdf]

Threshold-performance curve

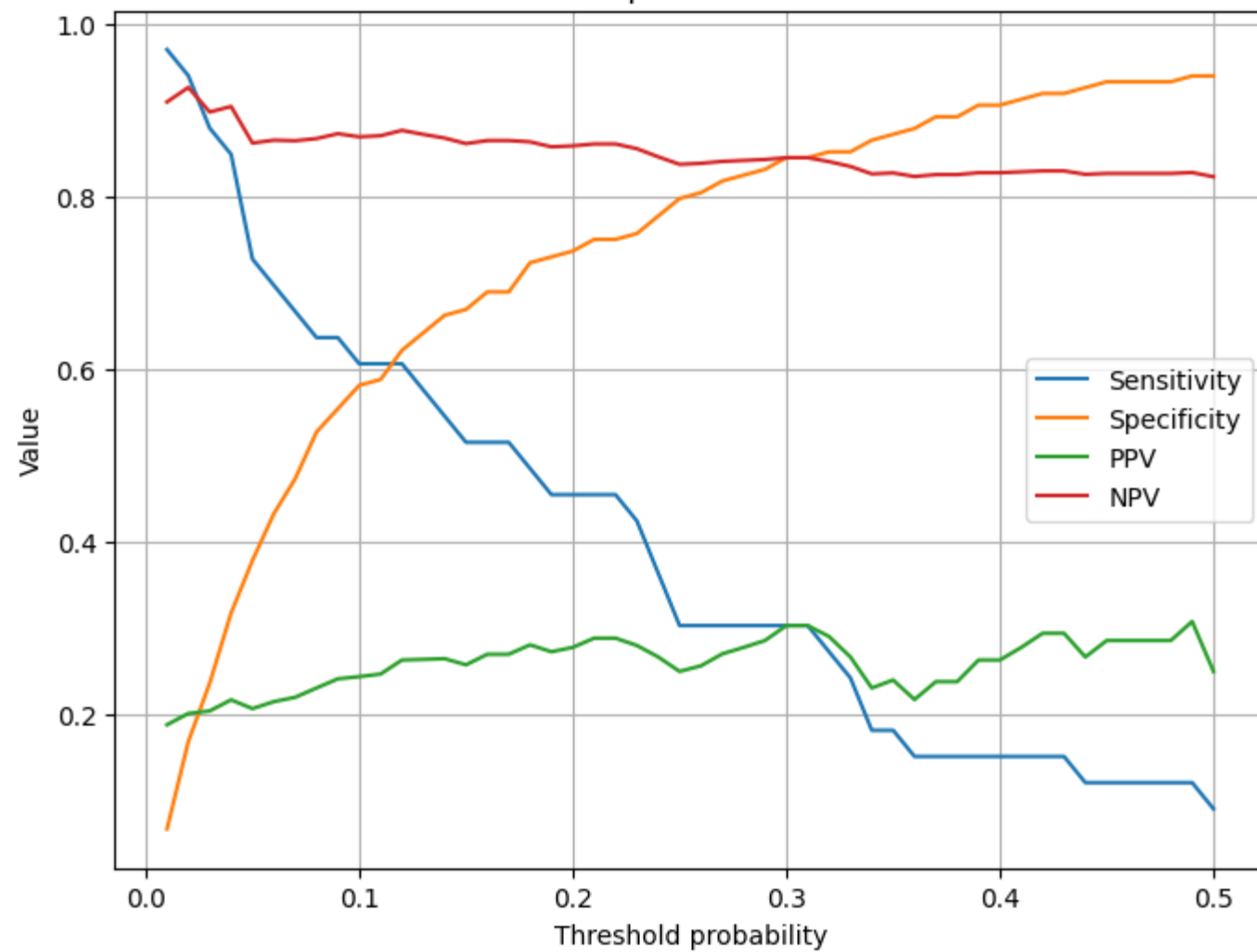

Supplement: Supplementary file 1 [file diagnostics-16-01578-s001.zip › Suuplementary Figure S2.pdf]

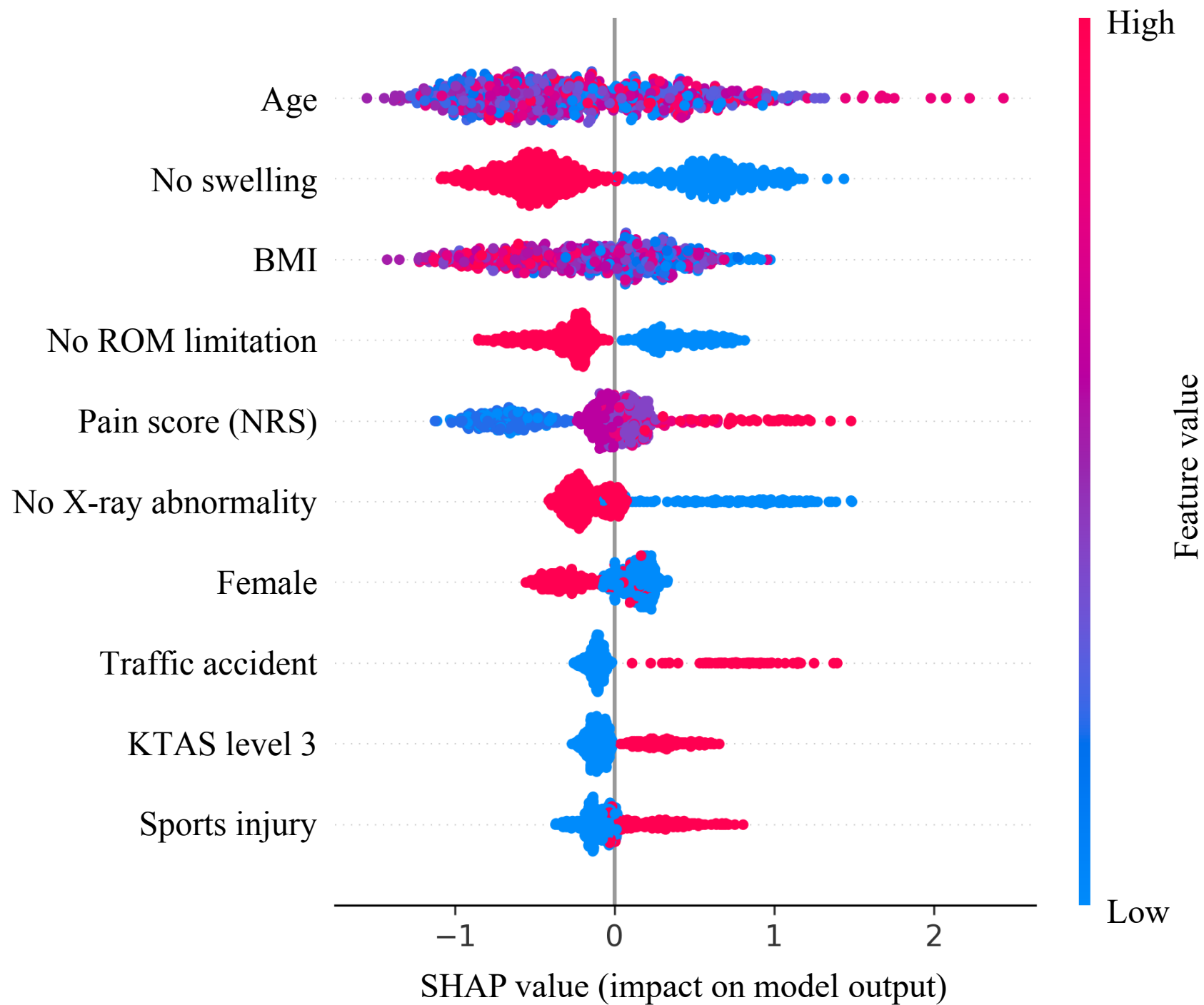

Supplement: Supplementary file 1 [file diagnostics-16-01578-s001.zip › Suuplementary Figure S3.pdf]
